# Dilated Dense U-Net for Infant Hippocampus Subfield Segmentation

**DOI:** 10.3389/fninf.2019.00030

**Published:** 2019-04-24

**Authors:** Hancan Zhu, Feng Shi, Li Wang, Sheng-Che Hung, Meng-Hsiang Chen, Shuai Wang, Weili Lin, Dinggang Shen

**Affiliations:** ^1^Department of Radiology and Biomedical Research Imaging Center, University of North Carolina at Chapel Hill, Chapel Hill, NC, United States; ^2^School of Mathematics Physics and Information, Shaoxing University, Shaoxing, China; ^3^Shanghai United Imaging Intelligence Co., Ltd., Shanghai, China; ^4^Department of Diagnostic Radiology, Kaohsiung Chang Gung Memorial Hospital, Chang Gung University College of Medicine, Kaohsiung, Taiwan; ^5^Department of Brain and Cognitive Engineering, Korea University, Seoul, South Korea

**Keywords:** fully convolutional network, dilated dense network, deep learning, hippocampal subfield segmentation, infant hippocampus

## Abstract

Accurate and automatic segmentation of infant hippocampal subfields from magnetic resonance (MR) images is an important step for studying memory related infant neurological diseases. However, existing hippocampal subfield segmentation methods were generally designed based on adult subjects, and would compromise performance when applied to infant subjects due to insufficient tissue contrast and fast changing structural patterns of early hippocampal development. In this paper, we propose a new fully convolutional network (FCN) for infant hippocampal subfield segmentation by embedding the dilated dense network in the U-net, namely DUnet. The embedded dilated dense network can generate multi-scale features while keeping high spatial resolution, which is useful in fusing the low-level features in the contracting path with the high-level features in the expanding path. To further improve the performance, we group every pair of convolutional layers with one residual connection in the DUnet, and obtain the Residual DUnet (ResDUnet). Experimental results show that our proposed DUnet and ResDUnet improve the average Dice coefficient by 2.1 and 2.5% for infant hippocampal subfield segmentation, respectively, when compared with the classic 3D U-net. The results also demonstrate that our methods outperform other state-of-the-art methods.

## Introduction

Hippocampus plays important roles in memory and spatial navigation, and is closely related to neurological diseases, such as autism, attention deficit hyperactivity disorder, and Alzheimer's Disease (Shi et al., 2009; Bartsch, 2012; Li et al., 2013). Hippocampus consists of several histologically and functionally specialized subfields (Dalton et al., 2017). It has been shown that different pathological conditions affect subfields differently, suggesting that subfields may provide more precise information for earlier disease diagnosis than simply using the whole hippocampus (Small, 2014).

Accurate segmentation of hippocampal subfields from magnetic resonance (MR) brain images is a critical step for studying memory-related neurological diseases. However, it is a challenging task especially in infant subjects, because of the small size of each hippocampal subfield, the blurred boundaries between subfields, and the large inter-subject variations. Manual segmentation is widely adopted, but it suffers high intra- and inter-operator variability, and is also excruciatingly time-consuming. Therefore, automatic hippocampal subfield segmentation methods are desirable. The existing automatic hippocampal subfield segmentation methods can be mainly categorized into three different types: (1) generative model based method (Van Leemput et al., 2009), (2) multi-atlas based method (Wang et al., 2013; Pipitone et al., 2014; Caldairou et al., 2016; Romero et al., 2017), and (3) multi-modality learning based method (Wu et al., 2018).

In the first category (Van Leemput et al., 2009), a generative model of image around the hippocampal area was produced by using a mesh-based probabilistic atlas learned from a set of ultra-high-resolution training images. The model was used to obtain automated hippocampal subfield segmentations on 10 adult subjects with the age range of 22–89 years.

In the past years, the second category of methods, namely multi-atlas based image segmentation (MAIS) methods, have been widely used in the field of medical image segmentation, including hippocampal subfield segmentation on adult subjects (Wang et al., 2013; Pipitone et al., 2014; Caldairou et al., 2016; Romero et al., 2017). In the MAIS methods, all selected atlas images are first registered to the target image, and the corresponding atlas labels are then warped to the target image space. Afterwards, these warped atlas labels are combined to obtain the final segmentation by label fusion. Note, in the MAIS methods, label fusion plays an important role. For example, a weighed voting label fusion was proposed (called joint label fusion) in a previous work (Wang et al., 2013), in which weights were obtained by minimizing the total expected error between the consensus segmentation and the ground-truth segmentation. This method was later combined with a learning-based error correction method for hippocampal subfield segmentation (Yushkevich et al., 2015). In another work (Romero et al., 2017), a new non-local patch based label fusion method was proposed based on a multi-contrast patch matching process. To further improve the segmentation, authors exploited a neural network-based error correction step for minimizing systematic segmentation errors. MAGeT-Brain (Multiple Automatically Generated Templates) was also proposed for automatic segmentation of the hippocampus and subfields, aiming to minimize the number of atlases needed whilst still achieving similar agreement to the multi-atlas approaches (Pipitone et al., 2014),. Besides, a surface patch-based segmentation method (Caldairou et al., 2016) was proposed for hippocampal subfield segmentation by combining surface-based processing with a patch-based template library and feature matching.

Besides the above two categories of methods, learning-based methods in the third category were also proposed for adult hippocampal subfield segmentation using 3T multi-modality MR images, including structural MRI (T1w, T2w) and resting-state fMRI (rs-fMRI) (Wu et al., 2018). In that paper (Wu et al., 2018), authors extracted both appearance features and relationship features to capture the appearance patterns in structural MR images and the connectivity patterns in rs-fMRI, respectively. These extracted features were then fed into a random forest classifier for voxel-wise classification.

Although several automatic methods have also been proposed for hippocampal subfield segmentation, most of them were evaluated only on the adult subjects, and thus cannot be directly applied to infant subjects due to insufficient tissue contrast and fast changing structural patterns of early hippocampal development.

In the recent years, deep convolutional neural networks (CNN) have been widely applied in the medical image segmentation (de Brébisson and Montana, 2015; Zhang et al., 2015; Moeskops et al., 2016). In CNN based segmentation methods, a patch centered at the target voxel (or pixel for 2D images) is taken as input for networks, and the tissue class of the center voxel is produced as the output of the networks. By learning sets of convolutional kernels, CNNs can capture highly non-linear mappings between inputs and outputs. Compared with MAIS methods and the traditional learning-based methods, CNN based segmentation methods are free of image registration and manual feature extraction.

A drawback of the CNN based segmentation approaches is that the input patches from neighboring voxels have huge overlap and the same convolutions are computed for many times. To address this limitation, fully convolutional networks (FCN) were proposed for voxel-wise dense prediction, by reformatting the fully connected layers as convolutional layers (Long et al., 2015). So far, a number of FCNs have been proposed and successfully used in medical image segmentation, including hippocampal segmentation (Ronneberger et al., 2015; Milletari et al., 2016; Chen Y. et al., 2017; Yu et al., 2017; Cao et al., 2018). For example, in the paper (Ronneberger et al., 2015), a U-net architecture was proposed by comprising a contracting (down-sampling) path, followed by an expanding (up-sampling) path. The features in the contracting path are concatenated to the corresponding features in the expanding path to recover the detailed image information that is lost during the down-sampling process. In the work (Milletari et al., 2016), authors extended U-net to a V-net structure by incorporating residual blocks (He et al., 2016a). In the paper (Yu et al., 2017), authors proposed a new volumetric convolutional neural network with mixed residual connections, where both the short connections between successive layers and the long connections between contracting path and expanding path are implemented with residual connections. In the work (Cao et al., 2018), authors proposed a multi-task CNN for joint hippocampal segmentation and clinical score regression with U-net as a subnet for hippocampal segmentation. In the paper (Chen Y. et al., 2017), authors proposed a multi-view ensemble approach to combine multiple decision maps obtained from several deep neural networks for hippocampal segmentation. Besides these contracting-expanding structures, dilated FCNs were also proposed for image segmentation, which can enlarge the receptive field exponentially without reducing any spatial resolution (Liang-Chieh et al., 2015; Yu and Koltun, 2015;Li et al., 2017; McKinley et al., 2017).

The U-net like structures are particularly successful in the field of medical image segmentation. One of the most important factors in the U-net is the long-skip connections which can concatenate the features in the contracting path to the corresponding features in the expanding path to recover the lost image information. However, the levels of features in the contracting path are much lower than those in the expanding path. Thus, it may not obtain optimal results when directly concatenating these features.

In this paper, we develop an automatic method to address the challenging infant hippocampal subfield segmentation problem with state-of-the-art deep learning techniques (LeCun et al., 2015; Litjens et al., 2017; Shen et al., 2017). To overcome the limitation of U-net structure, we propose a novel network by embedding a dilated dense network in the U-net, namely DUnet. The embedded dilated dense network can generate multi-scale features while keeping high spatial resolution, which is useful in fusing the low-level features in the contracting path with the high-level features in the expanding path. To further improve the performance, we use residual connections to group every pair of convolutional layers in DUnet, and obtain the Residual DUnet (ResDUnet).

The proposed method was applied for segmenting infant hippocampal subfields based on the Baby Connectome Project (BCP) dataset, containing 10 infant subjects. To the best of our knowledge, this is the first work to propose an automatic method for infant hippocampal subfield segmentation. To further illustrate the effectiveness of our proposed method, we also validated our proposed method for segmenting adult hippocampal subfields on a publicly available dataset. Experimental results show that our proposed DUnet and ResDUnet, respectively, improve the average Dice coefficient by 2.1 and 2.5% for infant hippocampal subfield segmentation, and 0.5 and 0.6% for adult hippocampal subfield segmentation, compared to the classic 3D U-net (Çiçek et al., 2016). Our proposed ResDUnet also outperforms both the state-of-the-art ConvNet (Yu et al., 2017) and hippocampal subfield segmentation method (HIPS) (Romero et al., 2017).

## Materials

Two image datasets were used for validating our method. The first dataset is from BCP, which was funded by the National Institutes of Health (NIH) as a component of the Lifespan Human Connectome Project. The BCP aims to provide scientists with unprecedented information about how the human brain develops from birth through early childhood and will uncover factors contributing to healthy brain development. For this project, researchers are acquiring MRI scans (including T1- and T2-weighted structural MRI, DTI, and rs-fMRI) of 500 typically developing children, ages 0–5 years, over the course of 4 years. In our experiment, 10 infant subjects (6 females/4 males) were randomly selected, each with T1w and T2w images acquired at 12 months old with 3T Siemens Prisma MRI scanners at the Biomedical Research Imaging Center (BRIC) at the University of North Carolina at Chapel Hill. Table 1 lists the imaging protocol for acquiring the T1w and T2w MR images. Five hippocampal subfields were manually labeled for each subject by the consensus of two neuroradiologists, including cornu ammonis sectors 1 (CA1), CA2/3, subiculum (SUB), CA4/dentate gyrus (DG), and Uncus. All T1w and T2w images underwent intensity inhomogeneity correction using the N3 bias field correction, and T2w images were rigidly aligned with corresponding T1w images. All images were aligned to a selected subject with affine registration.

**Table 1:** Imaging protocol for acquiring infant T1w and T2w MR images.

	**Matrix**	**FOV**	**Resolution mm^**3**^**	**FA**	**TE**	**TR**	**Slices orientation**	**AF/MB**	**Time**
T1w	320 × 320	256 × 256	0.8 × 0.8 × 0.8	8	2.24	2,400/1,060	208/Sag	AF = 2	6:38
T2w	320 × 320	256 × 256	0.8 × 0.8 × 0.8	VAR	564	3,200	208/Sag	AF = 2	5:57

**Figure 1:**
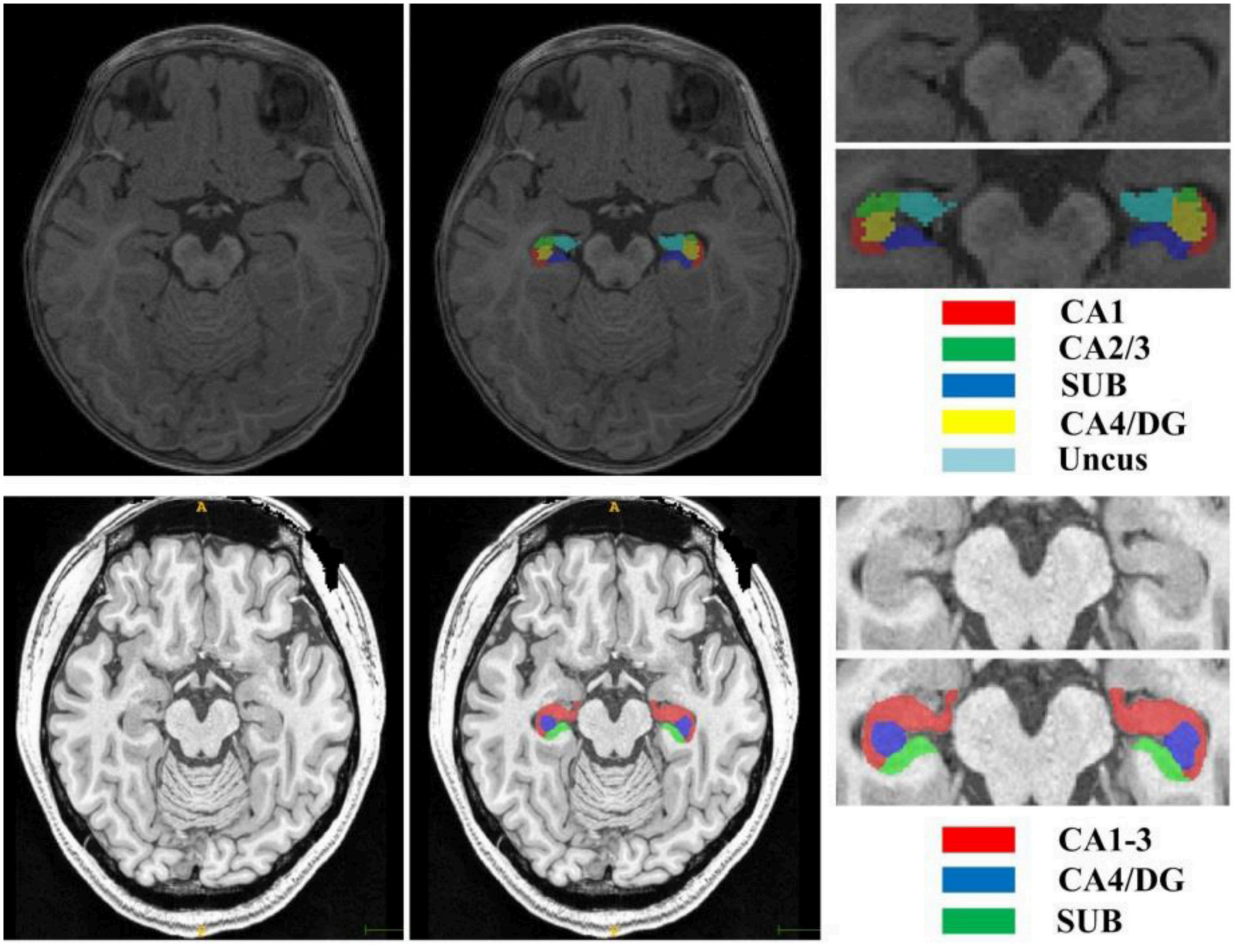
T1w image and manual segmentation of a representative subject from the BCP dataset **(top row)** and Kulaga-Yoskovitz dataset **(bottom row)**, respectively.

The second dataset is a publicly available dataset (https://www.nitrc.org/projects/mni-hisub25), which contains 25 adult subjects (31 ± 7 years, 12 males). Each subject consists of an isotropic 3D-MPRAGE T1-weighted image (TR = 3,000 ms; TE = 4.32 ms; TI = 1,500 ms; flip angle = 7°; matrix size = 336 × 384; FOV = 201 × 229 mm^2^; 240 axial slices with 0.6 mm slice thickness resulting in 0.6 × 0.6 × 0.6 mm^3^ voxels; acquisition time = 16.48 min), an anisotropic 2D T2-weighted TSE image (TR = 10,810 ms; TE = 81 ms; flip angle = 119°; matrix size = 512 × 512; FOV = 203 × 203 mm^2^, 60 coronal slices angled perpendicular to the hippocampal long axis, slice thickness of 2 mm, resulting in 0.4 × 0.4 × 2.0 mm^3^ voxels; acquisition time = 5.47 min), and a manually labeled image for hippocampal subfields including CA1-3, SUB, and CA4/DG (Kulaga-Yoskovitz et al., 2015). All T1w and T2w images underwent automated correction for intensity non-uniformity and intensity standardization. All images were linearly registered to the MNI152 space and resampled to a resolution of 0.4 × 0.4 × 0.4 mm^3^. Following the previous work (Romero et al., 2017), we named this dataset as Kulaga-Yoskovitz dataset. Figure 1 shows an example of T1w image and manual hippocampal subfield segmentation from the BCP dataset and the Kulaga-Yoskovitz dataset, respectively.

To facilitate the processing, we identified a bounding box that is big enough to cover the hippocampus of unseen target subject (Hao et al., 2014). In particular, for each subject in the BCP dataset and the Kulaga-Yoskovitz dataset, we went through all the training subjects to find the minimum and maximum *x, y, z* positions of the hippocampus, and empirically add 32 voxels in each direction as a bounding box to cover the hippocampus and its surrounding tissues. This step was done separately for these two datasets given the large hippocampus size differences in infants and adults. Then, we cropped all images with the box and applied a histogram matching method to the cropped images for obtaining similar intensity levels across all training subjects. To leverage the limited data, we *left-right* flipped each training image to double the number of training subjects.

## Methods

We propose a new FCN for hippocampal subfield segmentation. The FCN based segmentation methods can implement dense prediction by estimating the posterior probabilities for each voxel. Given the posterior probability *p*_*k*_(*x*|θ) of voxel *x* belonging to the *k*th category, where θ is the FCN model parameters, the hippocampal subfield label of voxel *x* is determined by

$$\begin{array}{l} L\left(x\right)=argmax_{k\in \mathbb{C}}\;p_{k}\left(x|\theta \right), \end{array}$$

where ℂ = {1, 2, …, *K*}, and *K* is the number of categories. In the remaining part of this section, we will introduce the details of our proposed FCN architectures and its loss function.

### Dilated Dense Network

**Figure 2:**
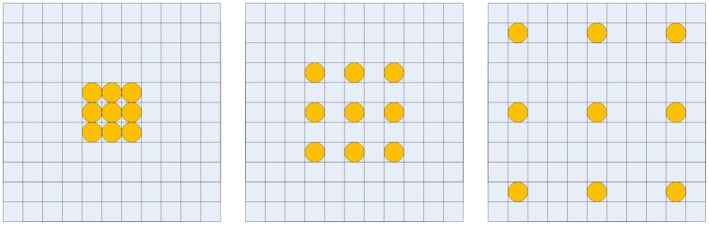
Illustration of dilated convolutional kernels: 1-dilated convolutional kernel **(left)**; 2-dilated convolutional kernel **(middle)**; 4-dilated convolutional kernel **(right)**.

Recent 3D neural networks often use small convolutional kernels with size 3 × 3 × 3 to reduce the number of parameters, and enlarge the receptive field by alternating convolutions and pooling operations to capture large image contexts (Çiçek et al., 2016). This successive down-sampling process will significantly reduce spatial resolution, which will lose detailed image information. Recently, dilated convolutions were proposed for semantic image segmentation (Liang-Chieh et al., 2015; Yu and Koltun, 2015). By using the dilated convolutions, the feature maps can be computed with a high spatial resolution, and the size of the receptive field can be enlarged arbitrarily. Figure 2 illustrates the dilated convolutional kernels with different dilation rates. Let *F*:ℤ^3^ → ℝ be a 3 dimensional discrete function, and *h*:Ω_*r*_ → ℝ be a discrete filter with a dilation rate *l*, where $\Omega_{r}={\left[-r,r\right]}^{3}⋂{\mathbb{Z}}^{3}$. The dilated convolution ^*^l can be defined as (Yu and Koltun, 2015),

(1) $$\begin{array}{l} \left(F{*}_{l}h\right)\left(p\right)=\underset{\text{s}+lt=p}{\sum }F\left(s\right)h\left(t\right). \end{array}$$

Note that, when *l* = 1, the dilated convolution becomes the normal convolution.

**Figure 3:**
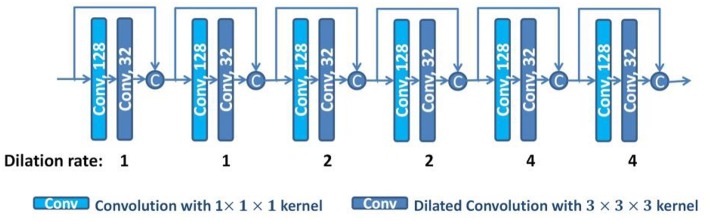
The structure of the dilated dense network. The number in each operation rectangle is the number of kernels. All operations are implemented in a 3D manner, and “c” denotes the concatenation.

With the dilated convolutions, we design a dilated dense network using dense connections (Huang et al., 2016), as shown in Figure 3. In the dilated dense network, we use dilated convolutions with different dilation rates to enlarge the receptive field, and use dense connections to concatenate all previous generated features to the current feature maps. To avoid overfitting, dropout operations are used after each 3 × 3 × 3 convolution with dropout rate 0.5 (Srivastava et al., 2014). Thus, the dilated dense network can capture contextual image information while keeping high spatial resolution and generate multi-scale image features. This dilated dense network will be embedded in our proposed DUnet, as introduced in the next subsection.

### Dilated Dense U-Net

**Figure 4:**
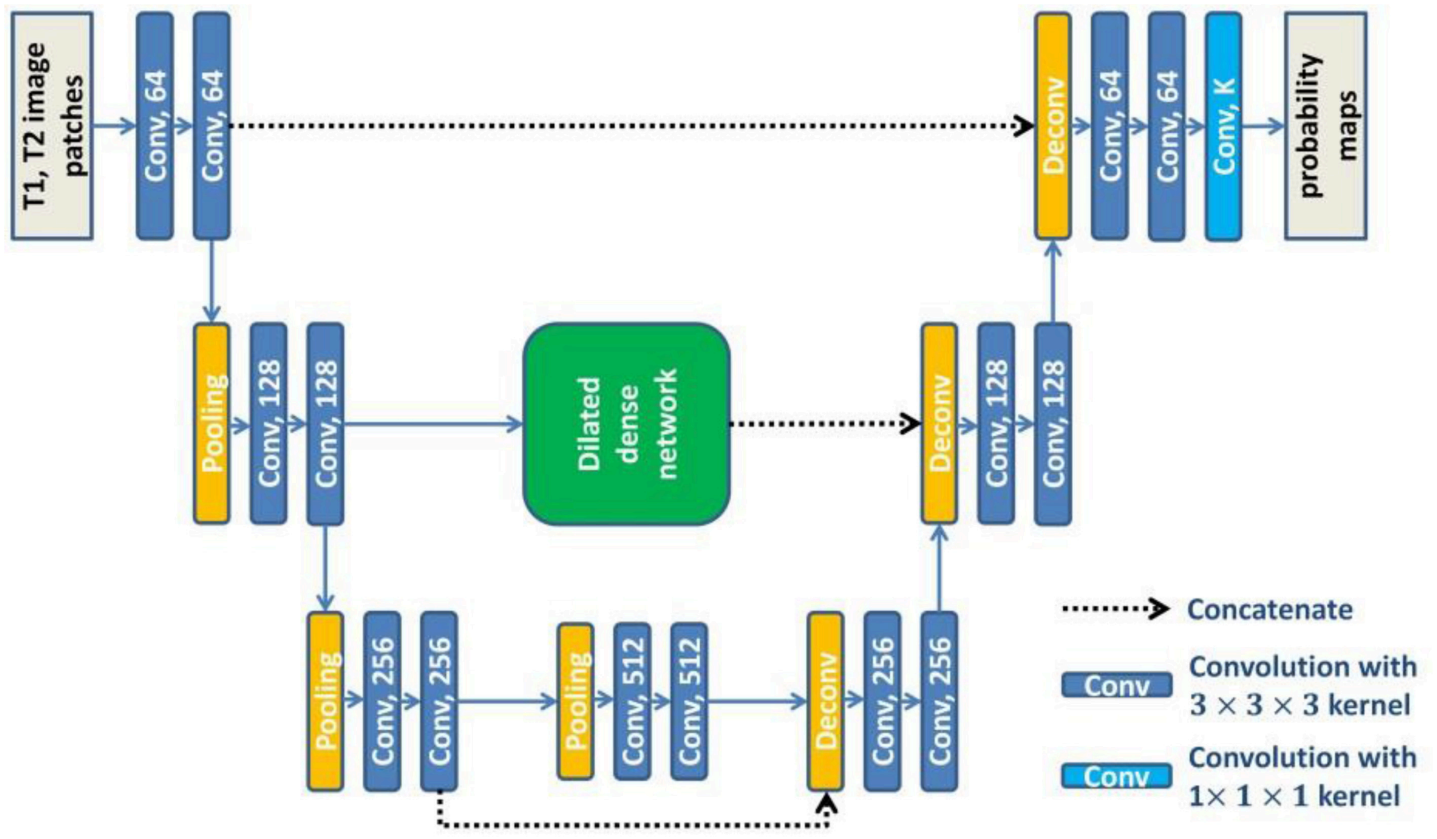
The structure of our proposed DUnet. The number in each operation rectangle is the number of kernels. All operations are implemented in a 3D manner.

U-net (Ronneberger et al., 2015) consists of a contracting path to extract abstract features and an expanding path to recover spatial resolution. The features in the contracting path are concatenated to the corresponding features in the expanding path to provide the detailed image information that is lost during the successive down-sampling steps. However, the level of features in the contracting path is much lower than that in the expanding path. It will not obtain the optimal results when directly concatenating these features. To overcome this limitation, we embed the dilated dense network in the U-net to obtain a new network (DUnet). Figure 4 shows the structure of our proposed DUnet.

Same to U-net, the proposed DUnet consists of a contracting path and an expanding path. The contracting path is built by alternating two 3 × 3 × 3 convolutions and one 2 × 2 × 2 max pooling operation with stride 2. The contracting path is followed by two 3 × 3 × 3 convolutions. Correspondingly, the expanding path is built by alternating one 4 × 4 × 4 deconvolution with stride 2, and two 3 × 3 × 3 convolutions. The expanding path is then followed by a 1 × 1 × 1 convolution, which outputs *K* feature maps (*K* is the number of label categories including the background). Each 3 × 3 × 3 convolution is followed by a batch normalization layer and a rectified linear unit (ReLU). Different from the original U-net, some padded convolution layers are also used to maintain the spatial dimension.

The feature maps before the first pooling layer and the last pooling layer are concatenated to the corresponding feature maps in the expanding path. The feature maps before the second pooling layer are first input into the dilated dense network which is introduced in the last subsection of this paper. Then, the output features of the dilated dense network are concatenated to the corresponding feature maps in the expanding path. The dilated dense network can provide multi-scale features while remaining high spatial resolution. Moreover, two different kinds of features provided by the dilated dense network and the contracting-expanding path are fused, providing more abundant image information for dense prediction.

### Residual Dilated Dense U-net

**Figure 5:**
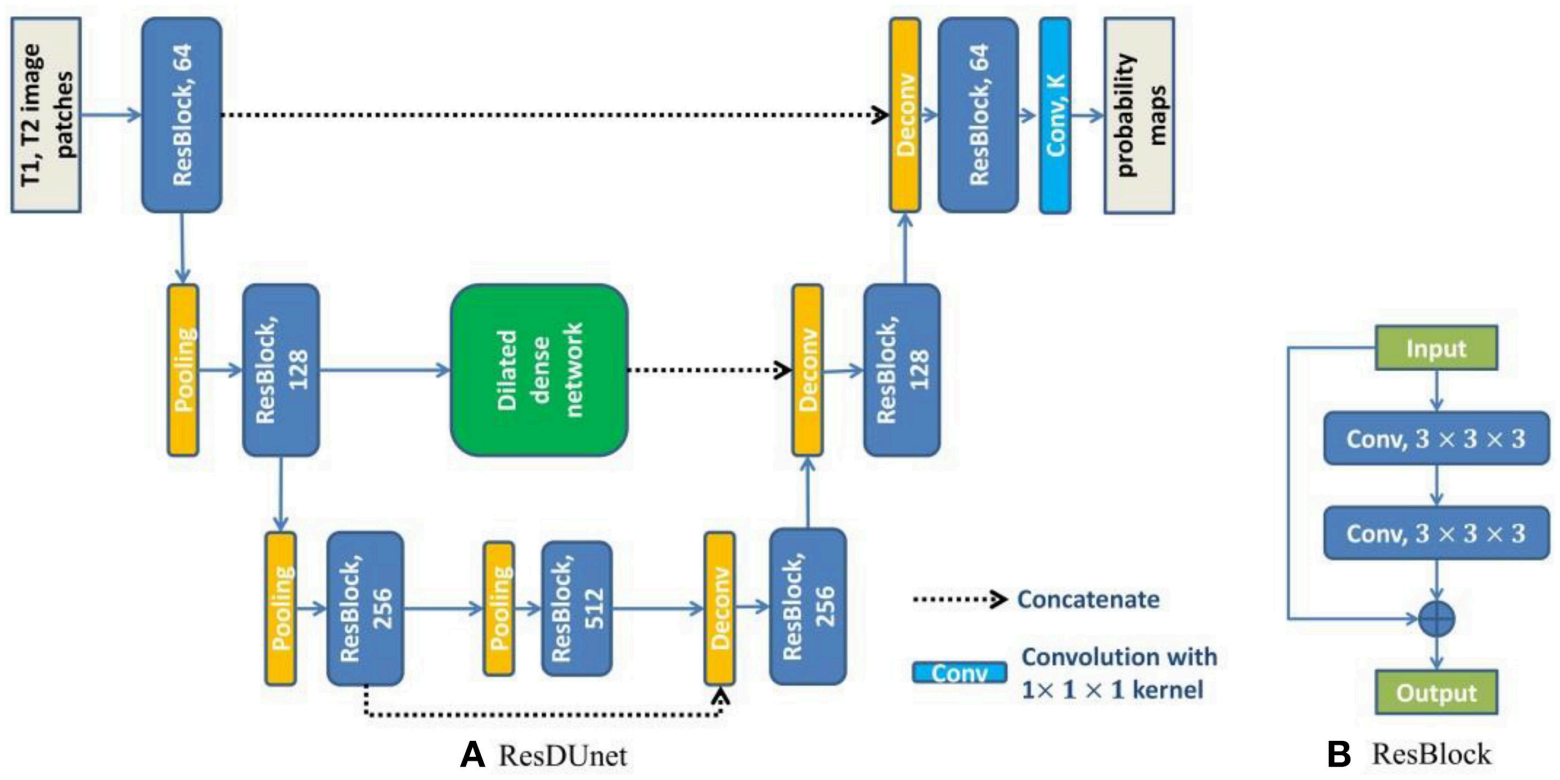
The structure of our proposed ResDUnet. The number in each operation rectangle is the number of kernels. “⊕” denotes the element-wise summation, and all operations are implemented in a 3D manner.

To further improve the performance, we use residual connections in DUnet to promote the information flow within the network (He et al., 2016a). Formally, the residual connection can be expressed as:

$$\begin{array}{l} x_{l}=H_{l}\left(x_{l-1}\right)+x_{l-1}, \end{array}$$

where *x*_*l*−1_ and *x*_*l*_ are the input and output of the *lth* unit, and *H*_*l*_(·) is a non-linear function which is used to learn the residual *x*_*l*_ − *x*_*l*−1_ of the *lth* unit. We group every pair of convolutional layers with one residual connection along the contracting path and the expanding path of DUnet, and obtain the Residual DUnet (ResDUnet). Figure 5 shows the structure of our proposed ResDUnet. The difference between ResDUnet and DUnet is the use of residual connections in ResDUnet, which connects two adjacent convolutions with an identity mapping (or a 1 × 1 × 1 convolution if the number of feature maps is not matched).

### Loss Function

We train our models using Softmax loss (Gu et al., 2017):

$$\begin{array}{l} L_{Softmax}=-\overset{N}{\underset{i=1}{\sum }}\overset{K}{\underset{k=1}{\sum }}1\left\{y_{i}=k\right\}\log\frac{e^{z_{k,i}}}{\sum_{j=1}^{K}e^{z_{j,i}}}, \end{array}$$

where *z*_*k, i*_ represents the *k*th output of the last network layer for the *ith* voxel, *y*_*i*_ ∈ {1, 2, …, *K*} represents the corresponding ground-truth label, *K* and *N* are the number of categories and the number of voxels, respectively. The term $\frac{e^{z_{k,i}}}{\overset{K}{\underset{j=1}{\sum }}e^{z_{j,i}}}$ represents the prediction probability for the *k*th class of the *ith* voxel, which is computed by the Softmax function.

### Evaluation Metrics

We evaluated the image segmentation results based on two types of segmentation evaluation measures (Jafari-Khouzani et al., 2011): Dice coefficient (Dice) and Average Symmetric Surface Distance (ASSD). Dice is used to measure the relative volumetric overlap between the automated segmentation and the manual segmentation, and ASSD is used to measure the agreement between segmentation boundaries. By denoting *A* as the manual segmentation, *B* as the automated segmentation, and *V*(*X*) as the volume of segmentation *X*, the two evaluation measures are defined as:

$$\begin{array}{l} \text{ Dice = 2}\frac{\text{V}(\text{A}\cap \text{B})}{V\left(\text{A}\right)\text{+V}(\text{B})},\;\\ \text{ASSD = }\left({\text{mean}}_{\text{e}\in \partial \text{A}}\left({\text{min}}_{\text{f}\in \partial \text{B}}\text{d}\left(\text{e},\text{f}\right)\right){\text{+mean}}_{\text{e}\in \partial \text{B}}\left({\text{min}}_{\text{f}\in \partial \text{A}}\text{d}\left(\text{e},\text{f}\right)\right)\right)\text{/2}, \end{array}$$

where ∂*A* denotes the boundary voxels of *A*, and *d*(·, ·) is the Euclidian distance between two points.

## Experiments and Results

### Experimental Details

Five-fold cross validation was used in the experiment for the BCP dataset. In each fold, we selected 7 subjects for training, 1 subject for validation, and 2 subjects for testing. Experiments were performed using a NVIDIA Titan Xp with 12 GB memory. Because of the restriction of limited training subjects and GPU memory, we randomly extracted patches from each training subject, instead of using the whole images as input for each network. We extracted about 1,300 patches from each subject. These patches were extracted as follows. First, we extracted patches one by one with stride of 2 × 2 × 2. The extracted patches that contain at least one hippocampal voxel were taken, and were numbered as 1, 2,…, *n*. Then, these numbers were randomly reordered. At last, we took the first half part of the reordered patches as our training patches. The patch size was optimally set to 24 × 24 × 24 by comparing the results obtained by the baseline 3D U-net method with different patch sizes, which is shown in Table 2. Since both T1w and T2w images were available, we concatenated the corresponding T1w and T2w image patches as input for each network. The networks were trained by Adam method with a batch size of 5, which were implemented with Caffe (Jia et al., 2014). The learning rates were initially set to 0.0001 and were decreased by a factor of γ = 0.1 every 10,000 iterations. We used a weight decay of 0.0005 and a momentum of 0.9 in all networks. The training process was stopped after 60,000 iterations. For segmenting a testing image, patches were extracted to feed into the trained models with an overlapped sliding windows strategy. The patch size was set to 24 × 24 × 24 with stride of 8 × 8 × 8. We used a majority voting strategy for the overlap regions to get the whole image prediction. Note that we used the same hyper-parameters during the 5-fold cross-validation.

**Table 2:** Mean (STD) values of Dice for each subfield segmentation using different patch sizes (*R*×*R*×*R*) on the BCP dataset by 3D U-net.

	***R* = 16**	***R* = 24**	***R* = 32**
CA1	0.635 (0.066)	**0.648** (0.078)	0.638 (0.107)
CA2/3	0.565 (0.071)	**0.567** (0.082)	0.556 (0.099)
SUB	0.717 (0.038)	**0.719** (0.080)	0.708 (0.123)
CA4/DG	**0.711** (0.063)	0.709 (0.072)	0.706 (0.057)
Uncus	0.710 (0.034)	**0.712** (0.050)	0.704 (0.069)
Average	0.668	**0.671**	0.662

*Higher Dice values indicate better segmentation performance. The best results are shown in bold*.

**Figure 6:**
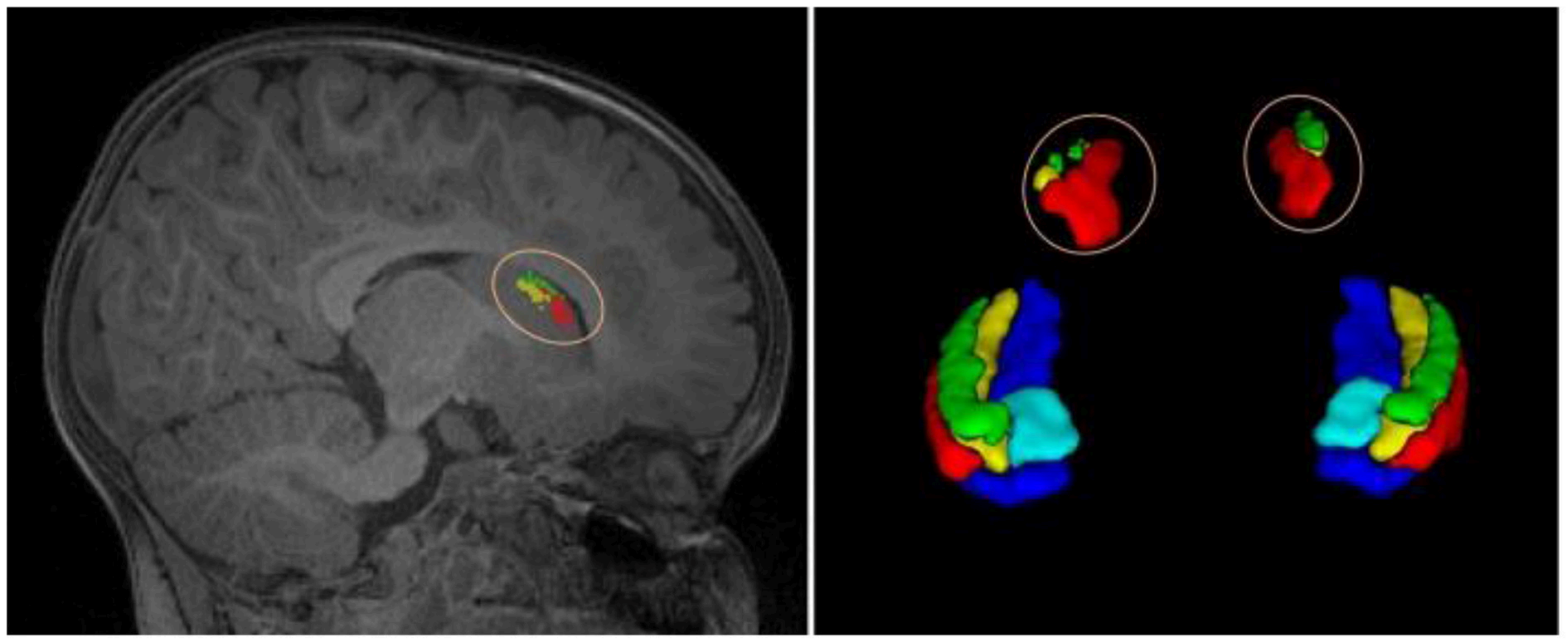
An example of isolated tiny blocks, outside the hippocampal region, appeared in the automated segmentation.

As the networks are trained based on image patches extracted around the hippocampus, the global spatial information of brain structures may not be perfectly captured. Thus, the obtained network models can well-recognize the hippocampal subfields around the hippocampus, but cannot recognize those far away from hippocampal region. For example, a patch in the caudate (denoted by the pink circle in the left of Figure 6) may look similar to the patches in the hippocampus, and will be classified to hippocampal subfields in the testing stage. As a result, there are some isolated false positives outside the hippocampal region, as shown in Figure 6. To remove these artifacts automatically, our post-processing steps include searching the voxels of each automated segmentation to find the non-zero neighbors of current voxel, and to obtain several connected regions. Then, we selected two regions with maximum volumes for the final left and right hippocampal subfields.

Five-fold cross validation was also used in the Kulaga-Yoskovitz dataset. In each fold, we selected 15 subjects for training, 5 subjects for validation, and 5 subjects for testing. The same experimental settings were used as the BCP dataset, except that the patch size was set to 32 × 32 × 32 as the resolution of images in this dataset is much higher, and the batch size was set to 3 because of the GPU memory limit. The same post-processing was used to remove isolated tiny blocks outside the hippocampal region.

### The Efficacy of Multi-Modality

In this subsection, we studied the efficacy of multi-modality by comparing the segmentation results obtained using only single modality images (i.e., T1w or T2w) and multi-modality images (T1w+T2w), respectively. All experiments were carried out on the BCP dataset with the same network architecture (ResDUnet) and the same training strategies. Table 3 lists the Dice coefficients of segmentation results using different image modalities. It shows that training using multi-modality images can obtain better results in the most subfields, compared with those using only either T1w or T2w single-modality images. This demonstrates that the network trained with multi-modality images can generate more discriminative features, which improves the performance of hippocampal subfield segmentation. From the results, we also find that T1w images can provide more useful information than T2w images for hippocampal subfield segmentation on the BCP dataset. In some subfields, training using only T1w images obtains similar or even a little better segmentation results than those using multi-modality images.

**Table 3:** Mean (STD) values of Dice for each subfield segmentation using different modalities on the BCP dataset.

	**T1w**	**T2w**	**T1w+T2w**
CA1	**0.674** (0.044)	0.604 (0.142)^*^^#^	0.672 (0.050)
CA2/3	0.571 (0.069)^*^	0.546 (0.104)^*^	**0.598** (0.041)
SUB	**0.745** (0.032)	0.644 (0.223)^*^^#^	0.745 (0.051)
CA4/DG	0.723 (0.027)	0.662 (0.157)^*^	**0.729** (0.032)
Uncus	0.725 (0.031)	0.645 (0.203)^*^^#^	**0.736** (0.035)
Average	0.688	0.620	**0.696**

*Higher Dice values indicate better segmentation performance. The best results are shown in bold*.

* Indicates that T1w + T2w achieves significant improvement over the corresponding method, and

# *indicates that T1w achieves significant improvement over the corresponding method in the Wilcoxon signed rank tests with p < 0.05*.

### Comparison With State-of-the-Art Methods

Our proposed method was also compared with two state-of-the-art networks, namely, 3D U-net (Çiçek et al., 2016) and ConvNet (Yu et al., 2017). The 3D U-net is extended from the previous 2D version (Ronneberger et al., 2015) into a 3D variant for volumetric feature representation. For a fair comparison, the 3D U-net used in our experiments consists of three pooling layers and three deconvolutional layers, which are the same as our proposed DUnet. The only difference is that the dilated dense network is used to fuse the middle level features of the contracting path with those of the expanding path in DUnet, instead of directly concatenating them as in 3D U-net. ConvNet (Yu et al., 2017) is a volumetric convolutional neural network with mixed residual connections, which also consists of three pooling layers and three deconvolutional layers. In ConvNet, residual connections are used between the successive convolution layers to form the residual blocks, and also between the feature maps of contracting path and those of expanding path. Besides, ConvNet (Yu et al., 2017) exploits a deep supervision mechanism to accelerate its convergence speed. All these comparative networks use Softmax loss as loss function, and the same post-processing is used to remove the tiny isolated blocks of segmentation results that appear outside of the hippocampal region.

**Figure 7:**
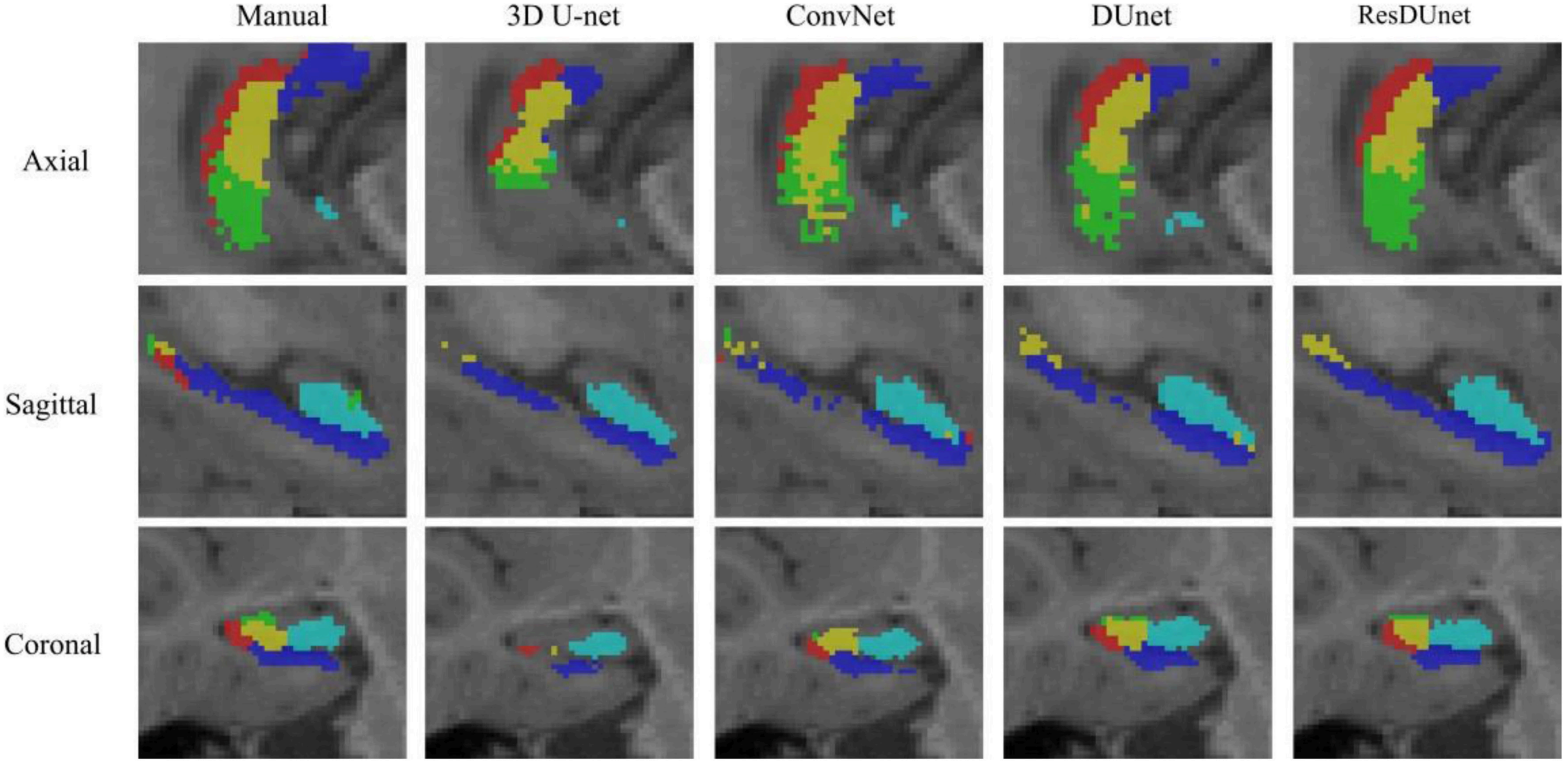
Hippocampal subfield segmentations of a randomly selected subject from the BCP dataset, obtained by manual segmentation, and four different networks.

Table 4 reports the Dice coefficients of the segmentation results obtained by different networks on the BCP dataset. It shows that our proposed DUnet outperforms 3D U-net (Çiçek et al., 2016) in segmenting CA1, SUB, CA4/DG and Uncus, and our proposed ResDUnet outperforms 3D U-net (Çiçek et al., 2016) in segmenting CA1, CA2/3, SUB, and Uncus, according to the Wilcoxon signed rank tests with *p* < 0.05. As can be seen in the table, our proposed ResDUnet achieves the highest Dice coefficient for the average of subfields. Table 5 reports the ASSD coefficients of the segmentation results, which shows that our proposed ResDUnet achieves the best ASSD coefficient for the average of subfields. Figure 7 shows hippocampal subfield segmentations of a randomly selected subject from the BCP dataset, obtained by manual segmentation and four different networks. It can be seen that our proposed ResDUnet achieves the most accurate results.

**Table 4:** Mean (STD) values of Dice for each subfield segmentation by different networks on the BCP dataset.

	**3D U-net (Çiçek et al., 2016)**	**ConvNet (Yu et al., 2017)**	**DUnet (proposed)**	**ResDUnet (proposed)**
CA1	0.648 (0.078)^*^^#^	0.670 (0.046)	0.665 (0.061)	**0.672** (0.050)
CA2/3	0.567 (0.082)^*^	0.584 (0.038)^*^	0.589 (0.045)^*^	**0.598** (0.041)
SUB	0.719 (0.080)^*^^#^	0.737 (0.045)^*^	0.742 (0.052)	**0.745** (0.051)
CA4/DG	0.709 (0.072)^#^	0.726 (0.030)	**0.733** (0.028)	0.729 (0.032)
Uncus	0.712 (0.050)^*^^#^	0.721 (0.035)	0.733 (0.034)	**0.736** (0.035)
Average	0.671	0.688	0.692	**0.696**

*Higher Dice values indicate better segmentation performance. The best results are shown in bold*.

* Indicates that ResDUnet achieves significant improvement over the corresponding method, and

# *indicates that DUnet achieves significant improvement over the corresponding method in the Wilcoxon signed rank tests with p < 0.05*.

**Table 5:** Mean (STD) values of ASSD for each subfield segmentation by different networks on the BCP dataset.

	**3D U-net (Çiçek et al., 2016)**	**ConvNet (Yu et al., 2017)**	**DUnet (proposed)**	**ResDUnet (proposed)**
CA1	0.175 (0.089)^*^	**0.146** (0.033)	0.158 (0.048)	0.147 (0.034)
CA2/3	0.211 (0.104)^*^	0.175 (0.020)	0.178 (0.028)^*^	**0.170** (0.025)
SUB	0.153 (0.073)^*^	**0.132** (0.030)	0.136 (0.040)	0.134 (0.039)
CA4/DG	0.157 (0.080)	**0.133** (0.019)	0.134 (0.019)	**0.133** (0.020)
Uncus	0.179 (0.055)^*^	0.168 (0.038)	0.170 (0.041)	**0.167** (0.044)
Average	0.175	0.151	0.155	**0.150**

*Smaller ASSD values indicate better segmentation performance. The best results are shown in bold*.

* *Indicates that ResDUnet achieves significant improvement over the corresponding method in the Wilcoxon signed rank tests with p < 0.05*.

### Results on a Public Adult Dataset

Tables 6, 7 list the Dice and ASSD coefficients of the segmentation results obtained by five different networks on the Kulaga-Yoskovitz dataset. The results show that our proposed DUnet outperforms 3D U-net (Çiçek et al., 2016) and ConvNet (Yu et al., 2017) in segmenting CA1-3 and SUB, and our proposed ResDUnet outperforms 3D U-net (Çiçek et al., 2016) and ConvNet (Yu et al., 2017) in segmenting all subfields, according to the Wilcoxon signed rank tests with *p* < 0.05. Table 6 also lists the comparison of our proposed method with the state-of-the-art hippocampal subfield segmentation method (HIPS), which obtained the best segmentation results on the Kulaga-Yoskovitz dataset so far (Romero et al., 2017). Note that, for a fair comparison, we use the published results of HIPS as reported in Romero et al. (2017). It shows that our proposed DUnet and ResDUnet also outperform HIPS method, especially for segmenting the CA4/DG subfield which is the most difficult task (Dalton et al., 2017). Figure 8 shows hippocampal subfield segmentations of a randomly selected subject from Kulaga-Yoskovitz dataset, obtained by manual segmentation and four different networks. It can be seen that our proposed DUnet and ResDUnet achieve the most accurate results.

**Table 6:** Mean (STD) values of Dice for each subfield segmentation by five different methods on the KULAGA-YOSKOVITZ dataset.

	**HIPS (Romero et al., 2017)**	**3D U-net (Çiçek et al., 2016)**	**ConvNet (Yu et al., 2017)**	**DUnet (proposed)**	**ResDUnet (proposed)**
CA1-3	0.916 (0.015)	0.916 (0.011)^*^^#^	0.918 (0.010)^*^^#^	0.919 (0.011)	**0.920** (0.011)
CA4/ DG	0.862 (0.034)	0.871 (0.021)^*^	0.870 (0.016)^*^	0.875 (0.020)^*^	**0.879** (0.020)
SUB	0.886 (0.021)	0.883 (0.016)^*^^#^	0.887 (0.018)^*^^#^	**0.890** (0.016)	0.888 (0.018)^#^
Average	0.888	0.890	0.892	0.895	**0.896**

*Higher Dice values indicate better segmentation performance. Best results are shown in bold*.

* Indicates that ResDUnet achieves significant improvement over the corresponding method, and

# *indicates that DUnet achieves significant improvement over the corresponding method in the Wilcoxon signed rank tests with p < 0.05*.

**Table 7:** Mean (STD) values of ASSD for each subfield segmentation by four different networks on the KULAGA-YOSKOVITZ dataset.

	**3D U-net (Çiçek et al., 2016)**	**ConvNet (Yu et al., 2017)**	**DUnet (proposed)**	**ResDUnet (proposed)**
CA1-3	0.065 (0.011)^*^^#^	0.064 (0.009)^*^^#^	**0.062** (0.009)	**0.062** (0.010)
CA4/DG	0.077 (0.014)^*^	0.079 (0.015)^*^	0.075 (0.015)^*^	**0.072** (0.014)
SUB	0.069 (0.013)^*^^#^	0.066 (0.013)^*^^#^	**0.064** (0.012)	0.065 (0.013)^#^
Average	0.070	0.070	0.067	**0.066**

*Smaller ASSD values indicate better segmentation performance. The best results are shown in bold*.

* Indicates that ResDUnet achieves significant improvement over the corresponding method, and

# *indicates that DUnet achieves significant improvement over the corresponding method in the Wilcoxon signed rank tests with p < 0.05*.

**Figure 8:**
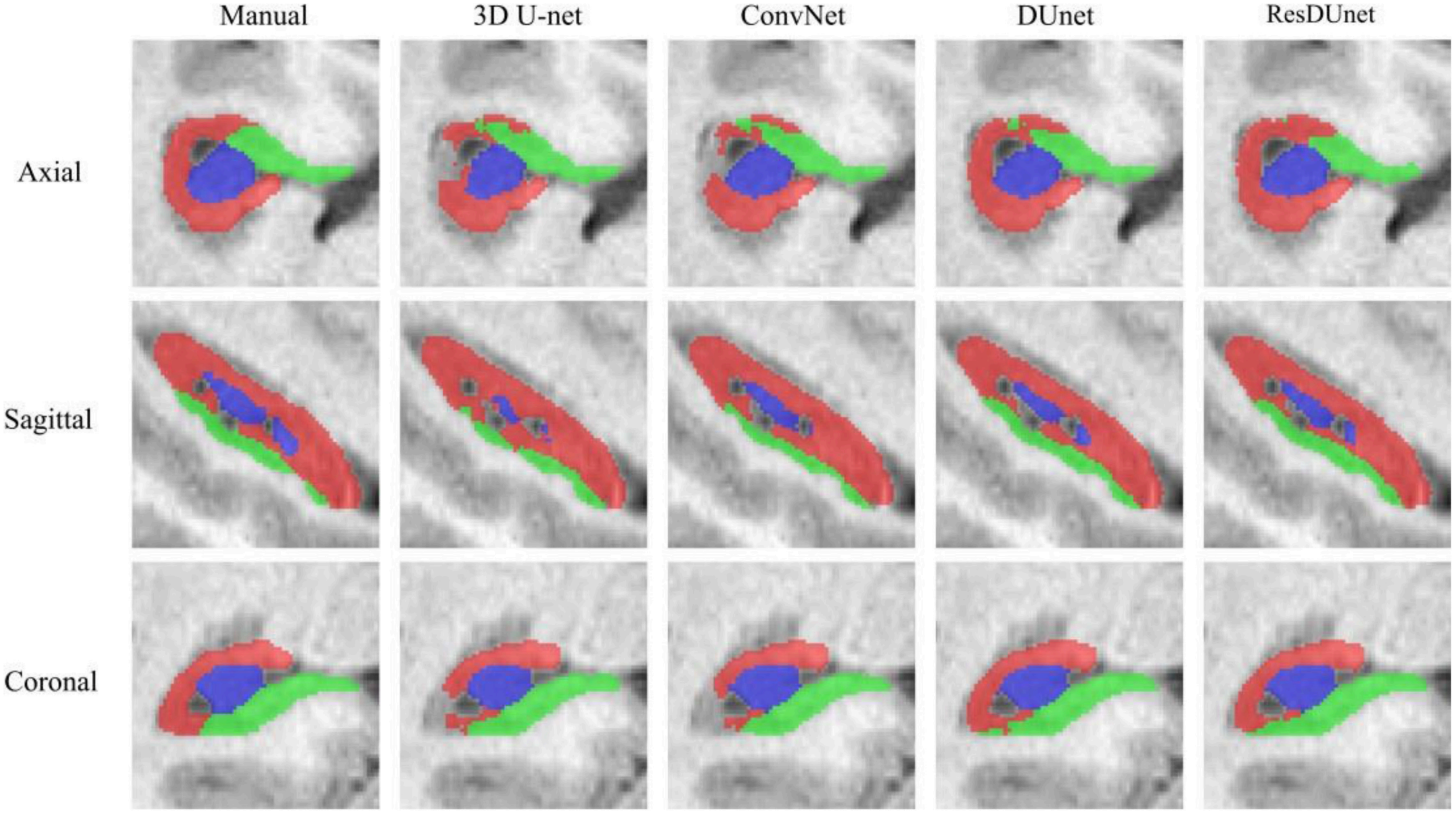
Hippocampal subfield segmentations of a randomly selected subject from the Kulaga-Yoskovitz dataset, obtained by manual segmentation, and four different networks.

## Discussion

FCNs have achieved great success in the field of medical image segmentation, which usually consist of a contracting path to extract abstract features, and an expanding path to up-sample the feature maps for dense prediction (Ronneberger et al., 2015; Çiçek et al., 2016; Chen H. et al., 2017; Lian et al., 2018; Nie et al., 2018). The detailed image information may be lost during these contracting and expanding processes. The existing U-net-like FCNs concatenate the feature maps in the contracting path to the corresponding feature maps in the expanding path to recover the lost image information. However, the levels of features in the contracting path are much lower than those in the expanding path. It may not obtain the optimal results when directly concatenating these features. To overcome this limitation and fully exploit multi-level image features, we proposed a new FCN by exploiting a dilated dense network to connect the features of the contracting path and the features of the expanding path. The dilated dense network uses the dilated convolutions to extract contextual features without reducing spatial resolution, and it also employs dense connections to aggregate multi-scale features. Thus, multi-scale features can be generated from the dilated dense network, which are fused with the corresponding features in the expanding path. To avoid overfitting, dropout operations are also used in the dilated dense network (Srivastava et al., 2014).

By using the dilated dense network to connect the feature maps in the contracting path and expanding path, our proposed method provides a way to fuse the finer-grained low-level features in the contracting path and the coarse high-level features in the expanding path. Moreover, the multi-scale features extracted by the dilated dense network are useful for segmenting multi-structures with different shapes and different scales. To further promote information propagation and accelerate the convergence, we introduce residual connections to group every pair of convolutional layers (He et al., 2016a,b).

Different from natural images, many imaging modalities are 3D in the field of medical image analysis. In the past few years, a lot of effort has been dedicated to exploit CNNs to process volumetric data. Some of them applied 2D CNNs to each slice of volumetric images (Prasoon et al., 2013; Setio et al., 2016; Chen Y. et al., 2017). To effectively make full use of the 3D spatial information, recent studies applied 3D CNNs to deal with volumetric images (Çiçek et al., 2016; Chen H. et al., 2017;Nie et al., 2018; Wachinger et al., 2018). Following these methods, our proposed FCNs were also implemented in a 3D manner. As the number of our training subjects is limited, we randomly extracted patches from each training subject, instead of using the whole image as the input for each network. The patch size was set to 24 × 24 × 24 for the BCP dataset and 32 × 32 × 32 for Kulaga-Yoskovitz dataset, considering different image resolutions in these two datasets.

As both T1w and T2w images were available for each subject, we concatenated the extracted T1w and T2w image patches as input to the networks. Compared with single modality data, multi-modality MR images can provide complementary contextual information, which contributes to better segmentation performance. From our experiments, we find that training using multi-modality images can obtain better results than using only single-modality images, and we also find that T1w images can provide more discriminative information than T2w images for hippocampal subfield segmentation.

Experimental results on the BCP dataset show that our proposed DUnet and ResDUnet improve the average Dice coefficient by 2.1 and 2.5%, respectively, for infant hippocampal subfield segmentation, compared with the 3D U-net (Çiçek et al., 2016). To further validate the effectiveness, we also applied our proposed method for adult hippocampal subfield segmentation based on a publicly available dataset. The results show that our proposed DUnet and ResDUnet improve the average Dice coefficients of 0.5 and 0.6%, respectively, compared with the 3D U-net (Çiçek et al., 2016). The improvement of our proposed ResDUnet method on both infant dataset and adult dataset comes from (1) multi-scale image features aggregation for distinguishing different hippocampal subfields; (2) utilization of the embedded dilated dense network for effectively fusing the low-level features in the contracting path and the high-level features in the expanding path; and (3) utilization of residual connections for promoting information propagation and accelerating the convergence.

However, the proposed method was mainly designed for infant hippocampal subfield segmentation on the BCP dataset. First, the embedded dilated dense network can provide multi-scale image features, which are especially useful for segmenting infant hippocampal subfields, since tissue contrast between infant hippocampal subfields are much blurrier than in adults. Second, the task of infant hippocampal subfield segmentation on the BCP dataset is to segment hippocampus into five parts (CA1, CA2/3, SUB, CA4/ DG, and Uncus), while there are only three parts (CA1-3, SUB, and CA4/DG) on the Kulaga-Yoskovitz dataset. Therefore, the segmented hippocampal subfields in the infant subjects are much smaller than those of the adult subjects. In our proposed network, the embedded dilated dense network can capture contextual image information without losing detailed image information, which is extremely useful for segmenting small structures.

## Conclusion

In this paper, we have proposed a new FCN by integrating U-net and dilated dense network for hippocampal subfield segmentation. Our proposed method can avoid losing the detailed image information in the successive down-sampling steps, effectively fuse the low-level features of the contracting path with the coarse high-level features of the expanding path, and generate multi-scale image features. Experimental results show that our proposed method outperforms the state-of-the-art methods.

## Ethics Statement

This study was approved by the Institutional Review Board in School of Medicine, the University of North Carolina (UNC) at Chapel Hill.

## Author Contributions

HZ, FS, LW, SW, and DS contributed to the method development and the manuscript preparation. WL contributed to providing infant imaging data, and S-CH and M-HC did manual labeling of infant hippocampal subfields. HZ undertook the statistical analysis.

### Conflict of Interest Statement

FS was employed by company Shanghai United Imaging Intelligence Co., Ltd., China. The remaining authors declare that the research was conducted in the absence of any commercial or financial relationships that could be construed as a potential conflict of interest.
